# Some Aspects of Intestinal Obstruction

**Published:** 1908-08-15

**Authors:** 


### Surgery.

### SOME ASPECTS OF INTESTINAL OBSTRUCTION.

### OPERATIVE TREATMENT (*continued*).

If the disease when found is in a freely movable portion of the intestine, its excision is not, as intestinal operations go, a difficult matter. The bowel is clamped on each side at a suitable distance from the proposed site of section—about three inches is enough to give the operator the necessary room; it is then cut across transversely on either side of the growth. If the mesentery is not involved, it is unnecessary to cut out a V-shaped portion. The cut ends of the gut may be united in a variety of ways. Simple suture is eminently the most satisfactory method in suitable cases. Two continuous layers of silk are inserted—a first through and through layer, and a continuous Lembert suture outside this. Cheyne and Burghard advise that the continuous sutures should be interrupted once or twice to avoid the risk of puckering up the stitch, and so diminishing the lumen of the bowel. Special attention must be paid to the exact adaptation of the two cut edges of the mesentery.

Mayo Robson's decalcified bone bobbin is especially useful in those cases where the gut above the stricture is much dilated and its lumen is consequently greater than that of the lower end, because when the bobbin is inserted it is possible to draw the sutures as tight as is necessary without fear of constricting the gut. The exact coaptation of two portions of gut whose lumens are widely unequal is very difficult if the method of simple suture be employed.

The use of Murphy's button has undeniable advantages, but it has also great drawbacks. Its use is specially indicated when the condition of the patient is such that rapidity of operation is essential, for its employment does undoubtedly save time. Its great disadvantages are that it narrows the intestine at the point of suture, and the button itself may cause an obstruction or give rise to ulceration as it passes through the intestine.

The excision of tumours in portions of the bowel which are not completely surrounded by peritoneum is generally extremely difficult. The operation has to be carried out in the depths of the wound, and accurate union of the posterior wall of the bowel is often impossible. Moreover, it may be found after excising the mass that the ends cannot be approximated at all. Where this is likely to happen or where the disease is so extensive that it cannot be excised, two alternatives confront the surgeon—a lateral anastomosis to short circuit the stricture and the formation of an artificial anus.

Of these two the former is infinitely preferable, and should be done wherever possible. The actual technique of lateral anastomosis does not differ from that of gastro-enterostomy. It is important to make a sufficiently large opening and to cut away any mucous membrane that protrudes, as the artificial opening shows a great tendency to close. An artificial anus should only be made when anastomosis is impossible, and never in the small intestine, except in extreme cases, because life can only be supported under these conditions for a few weeks. Unfortunately, most operations for intestinal obstruction are performed when the obstruction is complete, and although theoretically the same three alternatives are open to the surgeon, it is generally necessary in practice to relieve the symptoms as quickly as possible if the life of the patient is to be saved. It is in these cases that a diagnosis of the exact site of the lesion is so vitally important, since it is obviously worse than useless to perform a left inguinal colostomy if the stricture is situated above the sigmoid. There is much to be said in favour of doing a temporary cæcostomy as a routine measure in these cases, the argument being that malignant stricture is far more common in the large than in the small intestine, and that unless there is positive evidence that the disease is situated in the small intestine a cæcostomy should always be performed as a preliminary measure. An incision is made as for appendicitis, through which the presenting part of the distended cæcum can be stitched to the edges of the wound and opened at once with a Paul's tube if necessary. The operation is at once speedy and effective, and the question of excising the stricture or of doing a lateral anastomosis can be considered at a later date, when the patient has recoverd from the effects of the obstruction. This is a far better operation than a right inguinal colostomy, which is at the best of times a difficult operation, and in the distended state of the ascending colon may be quite impossible. If a second operation is done later, it is usually quite easy to close the cæcostomy opening; sometimes, however, this presents a little difficulty, and it may be necessary to make two or even more attempts before success is attained.

If it can be clearly demonstrated that the growth is in the rectum or the sigmoid, and if the surgeon considers that the condition is too far advanced for radical operation, then the proper treatment is left inguinal colostomy as a permanent measure. The operation is a simple one. A short incision is made, having its centre at McBurney's spot on the left side. The abdomen is opened by the muscle splitting method, and the sigmoid is brought into the wound. The gut is pulled gently downwards until the upper end will not come down any further. This avoids the risk of any subsequent prolapse. The mesentery is then pierced at a point where there are no veins with a Spencer-Wells' forceps, which is left *in situ* and fixed in position by shutting the blades on a piece of gauze. Two sutures are put in to steady the gut at each end, and the operation is complete. The gut can be opened next day or at the time of operation with a Paul's tube if the symptoms are urgent. The forceps is removed in from eight to ten days, and an admirable spur is usually obtained. The operation is, of course, only a palliative measure, but it allows the patient to live for a year, or even two, in comparative comfort.

